# Drug exposure definitions reported in real-world evidence studies on oncology medicines in patients with colorectal cancer: a scoping review

**DOI:** 10.1016/j.esmorw.2026.100750

**Published:** 2026-08-10

**Authors:** J.E.M. van Dommelen, H. Gardarsdottir, T.C.G. Egberts, T.J. Giezen

**Affiliations:** 1 Department of Clinical Pharmacy, Spaarne Gasthuis Hospital, Haarlem/Hoofddorp, The Netherlands; 2 Division of Pharmacoepidemiology and Clinical Pharmacology, Utrecht Institute for Pharmaceutical Sciences, Utrecht University, Utrecht, The Netherlands; 3 Department of Clinical Pharmacy, University Medical Centre Utrecht, Utrecht, The Netherlands; 4 Department of Pharmaceutical Sciences, University of Iceland, Reykjavik, Iceland

**Keywords:** colorectal cancer, oncological drug exposure, real-world evidence studies, scoping review

## Abstract

**Background:**

Defining, measuring, and reporting exposure in real-world data are important for the valid generation and interpretation of real-world evidence (RWE). This is especially challenging for oncology medicines, due to complicated treatment regimens and adjustments in dose and/or schedule. This scoping review aims to assess the reporting of drug exposure definitions for oncology medicines in RWE studies, using colorectal cancer (CRC) as a case study.

**Methods:**

We searched PubMed and Embase for RWE studies assessing clinical outcomes of cyclically administered oncology medicines in patients with CRC, published in 2023-2024. We extracted publication characteristics, study characteristics, and exposure variables. We calculated a drug exposure reporting score (0-7), composed of active substance, dose, dosing schedule, number of cycles, treatment episode length, and drug exposure start/end. We exploratively compared characteristics of studies with a high and low score.

**Results:**

Of the 61 included studies, 59% used hospital data, and 36.1% used drug administration data. Most studies (83.6%) reported the start of drug exposure, and 34.4% reported the end of drug exposure. Median drug exposure reporting score was 3.0 (interquartile range 2.0-4.0). Studies with a high score were 4.6 times more often published in an oncology journal and reported use of guidelines for observational studies 1.8 times more often than studies with a low score.

**Conclusion:**

Definitions of drug exposure are often underreported in RWE studies on oncology medicines. Lack of clarity concerning oncological drug exposure complicates the assessment of the methodological quality and reproducibility and may affect the interpretation of the association between oncology medicines and clinical outcomes.

## Highlights

• Drug exposure definitions are underreported in RWE studies in CRC.
• Drug exposure reporting score (0-7) captures seven oncological drug exposure variables.
• The median drug exposure reporting score in our study was 3.0 (IQR 2.0-4.0).
• Underreporting challenges the assessment of methodological quality and reproducibility.

## Introduction

Randomized clinical trials (RCTs) are considered the gold standard for generating evidence on the efficacy and safety of new medicines and for supporting marketing authorization decisions.^1^ At the time of approval, knowledge about the benefit–risk profile of new medicines is often incomplete. Differences in care settings, patient characteristics, treatment adherence, and outcome assessment imply that treatment effects observed in RCTs may not fully translate to clinical practice.^2^^,^^3^ This inherently results in residual uncertainty, which demands further evaluation of the positive and negative effects of medicines when used in routine clinical practice. This can be accomplished through real-world evidence (RWE) studies, which rely on real-world data (RWD) routinely collected in health care systems.

Uncertainty at the time of marketing authorization is particularly pronounced for oncology medicines.^4^ In 2024 and 2025, the European Medicines Agency (EMA) recommended authorization for 84 new active substances, of which 27 (32.1%) were oncology medicines.^5^^,^^6^ Oncology medicines are frequently approved under conditions of additional residual uncertainty, reflecting the high unmet medical need and the urgency to provide patients with timely access to new therapies. For example, oncology medicines can receive conditional marketing authorization when their anticipated benefits outweigh known and potential risks, despite remaining uncertainties.^1^^,^^7^ In 2024, three oncology medicines received conditional approval, increasing to six in 2025.^5^^,^^6^

Conditional approvals often rely on interim analyses, single-arm trials, or surrogate endpoints, such as progression-free survival (PFS) rather than overall survival. Innovative trial designs, such as single-arm trials with external controls, are increasingly used when conventional RCTs are infeasible due to small patient populations or the absence of appropriate control groups.^4^^,^^8^ However, these designs introduce methodological uncertainties. Additional uncertainty may occur because improved surrogate outcomes in oncology RCTs do not always translate into proportionally improved real-world clinical outcomes in observational studies.^9^

These uncertainties underscore the importance of reassessing the added benefit of costly oncology medicines, especially compared with other therapeutic areas, after approval.^10^ Indeed, 41% of oncology medicines (particularly those conditionally authorized) authorized by the EMA between 1995 and 2020 were later found to have a negative or nonquantifiable added benefit.^7^ In this context, RWE studies play a critical role in re-evaluating treatment effectiveness and safety and long-term benefit–risk assessment, thereby informing regulatory decision making, reimbursement, and clinical guideline development.^3^^,^^11^

Reflecting the growing importance of RWE in oncology, the European Society for Medical Oncology (ESMO) published the Guidance for Reporting Oncology Real-World Evidence (GROW) in 2023.^12^ This guidance emphasizes the need for transparent reporting of key methodological elements in RWE studies to minimize misclassification and bias. This includes the clear identification of exposure to systemic anticancer treatment (SACT) and the measurement of clinical outcomes. Outcome definitions and assessments are highly standardized and protocol-driven in RCTs, whereas they are shaped by routine clinical practice in observational studies. This leads to potential differences in outcome measurement.^4^ A recent publication highlighted that RWE studies often define, measure, and report PFS less explicitly than RCTs.^13^ This finding reinforces the need for improved methodological rigor. By analogy, exposure to oncology medicines is also likely to be less clearly defined and reported in RWE studies than in RCTs.

At present, ESMO GROW does not provide concrete guidance on how exposure to oncology medicines should be defined and reported in RWE studies. Measuring drug exposure in oncology is inherently more complex than for many other (chronic) medicines, due to its multidimensional nature. Oncology treatments often involve varying individual doses, combinations of multiple agents, different routes of administration (e.g. oral and parenteral), repeated cycles over time, treatment interruptions, and/or dose modifications. These characteristics complicate exposure definition and increase the risk of heterogeneity, misclassification, and bias in observational research.

Against this background, the objective of this scoping review was to assess how drug exposure to oncology medicines is defined and reported in RWE studies. For feasibility and illustrative purposes, the scope was restricted to patients with (metastatic) colorectal cancer [(m)CRC].

## Methods

We conducted this scoping review according to the Preferred Reporting Items for Systematic Reviews and Meta-Analyses Extension for Scoping Review 2018^14^ and the methodological guidance on conducting scoping reviews by the Joanna Briggs Institute.^15^

### Search and selection strategy

Our focus was on observational RWE studies (i.e. cohort, case–control, or case–crossover design^16^), which aimed to assess the effectiveness and/or safety of cyclically administered oncology medicines in patients with (m)CRC. We systematically searched the bibliographic databases PubMed and Embase to identify eligible RWE studies published until January 2025. We included RWE studies published in a scientific journal in the English language if the full text was available. We restricted the inclusion to RWE studies published in 2023 and 2024 because we considered recent years more relevant for today’s decision making and clinical practice. We excluded RWE studies if the determinant was a treatment other than solely SACT (i.e. surgery, radiotherapy, ablation, hyperthermic intraperitoneal chemotherapy, hepatic arterial infusion pump chemotherapy, or radioembolization). Not all RWE studies explicitly mention study design in the title/abstract. Therefore, we added terms related to observational data sources (e.g. dispensing data) to the search string to complement the systematic search with RWE studies retrospectively using routinely and prospectively documented health care data.^16^ The full search string can be accessed in Supplementary Table S1, available at https://doi.org/10.1016/j.esmorw.2026.100750. The initial search was carried out in July 2024 and repeated in July 2025.

We deduplicated the search output and screened the titles and abstracts of retrieved records with the aid of ASReview. ASReview is an open-source machine learning tool developed at Utrecht University, in which four phases of active learning accelerate the selection of relevant scientific literature.^17^ In the first phase, one reviewer (J.E.M.v.D) screened the titles and abstracts of a randomly selected 1% of the search output for relevance. The first model used this subset to train itself. The first round of prioritization stopped when at least 10% of the search output had been screened, and 50 consecutive records were marked irrelevant. The second active learning model stopped prioritizing when 50 consecutive records were marked as irrelevant. The reviewer screened title/abstracts of the prioritized records and the full text of the relevant RWE studies and discussed screening uncertainties with two reviewers (H.G. and T.J.G.), until we reached consensus in case of discrepancies.

### Data extraction

One author (J.E.M.v.D) extracted data, primarily from the methods section of the included RWE studies or referenced published protocols and/or previously published RWE studies. The predefined data extraction form can be accessed in Supplementary Table S2, available at https://doi.org/10.1016/j.esmorw.2026.100750. The two reviewers (H.G. and T.J.G.) validated the data extraction for a random sample. Once again, the first reviewer (J.E.M.v.D.) discussed extraction uncertainties with the other two reviewers (H.G. and T.J.G.) until we reached consensus.

We selected the following seven variables as key components necessary to construct an oncological treatment episode: reporting (or deduction from abbreviated regimen names) of active substance; start of drug exposure; dose; dosing schedule; number of cycles; length of treatment episode; and end of drug exposure. These key components were selected after thorough discussion among the authors, inspired by the guideline ‘The reporting of studies conducted using observational routinely collected health data statement for pharmacoepidemiology’.^3^

We also extracted additional variables contributing to oncological drug exposure: regimen; route of administration (e.g. intravenous and oral); treatment switch; dose adjustment; cycle prolongation; and bridging. Table 1 can be consulted for examples.

**Table 1:** Examples of variables contributing to oncological drug exposure^18^, ^19^, ^20^, ^21^, ^22^

Variable	Example
Key components of oncological drug exposure	
Active substance	
Reported or deducible from regimen	… trifluridine/tipiracil… e.g. FOLFOX consists of 5-fluorouracil, folinic acid, and oxaliplatin
Start of drug exposure	… start dates … were collected … …the time from trifluridine/tipiracil start …
Dose	The initiating dose of trifluridine/tipiracil treatment was 35 mg/m^2^
Dosing schedule	… twice daily, 5 days of treatment followed by a 2-day rest period each week for 2 weeks and then a 14-day rest period (28-day cycle)
No. of cycles	… number of cycles … were collected …
Treatment episode length	Treatment duration was defined as time from index date to last administration of bevacizumab-awwb or bevacizumab reference product …
End of drug exposure	… stop dates, … treatment discontinuation and reasons … were collected …
Additional variables contributing to oncological drug exposure	
Regimen	… including FOLFOX, FOLFIRI, FOLFOX/FOLFIRI + bevacizumab, FOLFOX/FOLFIRI + cetuximab, or FOLFOX/FOLFIRI + panitumumab
Route of administration	Patients received TAS-102 by twice daily oral administration and/or bevacizumab by single 30-minute i.v. infusion …
Treatment switch	… initiation of the subsequent line of therapy …
Dose adjustment	… dose modifications … were collected …
Cycle prolongation	… administration postponements … were collected …
Bridging	… including any treatment interruptions or other breaks (no >90 days in length)

FOLFOX, 5-fluorouracil/folinic acid/oxaliplatin; FOLFIRI, 5-fluorouracil/folinic acid/irinotecan; i.v., intravenous; TAS-102, trifluridine/tipiracil.

Finally, we extracted publication and study characteristics: first author; title; year of publication; journal; journal category (e.g. oncology); journal quartile (as of 2024, extracted from the SCImago Journal and Country Rank^23^); open access status; journal impact factor (IF) (as of 2024); number of citations (on 19 December 2025); continent in which the RWE study was conducted; clinical outcomes (e.g. effectiveness and safety); setting (e.g. primary care and hospital); data source (e.g. administrative data source and hospital data source); reported use of guidelines for observational studies [e.g. Strengthening the Reporting of Observational Studies in Epidemiology guideline]; exposure-related inclusion criterion (e.g. number of administrations and number of cycles); number of included patients; start of follow-up definition (e.g. diagnosis and exposure); follow-up time in months; origin of exposure (e.g. drug prescription data, drug dispensation data, and drug administration data); treatment type (e.g. monotherapy and combination therapy); and type of SACT [e.g. chemotherapy and targeted therapy (i.e. small molecules and monoclonal antibodies that target specific biomarkers or receptors, respectively^24^)].

### Data analysis

We descriptively summarized the characteristics of the included RWE studies and the reporting of key components of oncological drug exposure in percentage (count) or median [interquartile range (IQR)]. Subsequently, we calculated a drug exposure reporting score for the key components of oncological drug exposure. For the reporting of each key component, a value of 1 was added to the drug exposure reporting score. The partial reporting of active substance, dose, dosing schedule, or number of cycles added a value of 0.5 to the drug exposure reporting score. This all adds up to a maximum attainable score of 7. We exploratively compared publication and study characteristics (i.e. journal category, journal quartile, open access status, journal IF, number of citations, and the reported use of guidelines for observational studies) and calculated their relative risks (RRs) and 95% confidence intervals (CIs) for RWE studies within the highest versus lowest quartile of the drug exposure reporting score. We used R software, version 4.3.3 (R Foundation for Statistical Computing, Vienna, Austria), to perform all statistical analyses.

## Results

### Study selection

**Figure 1:**
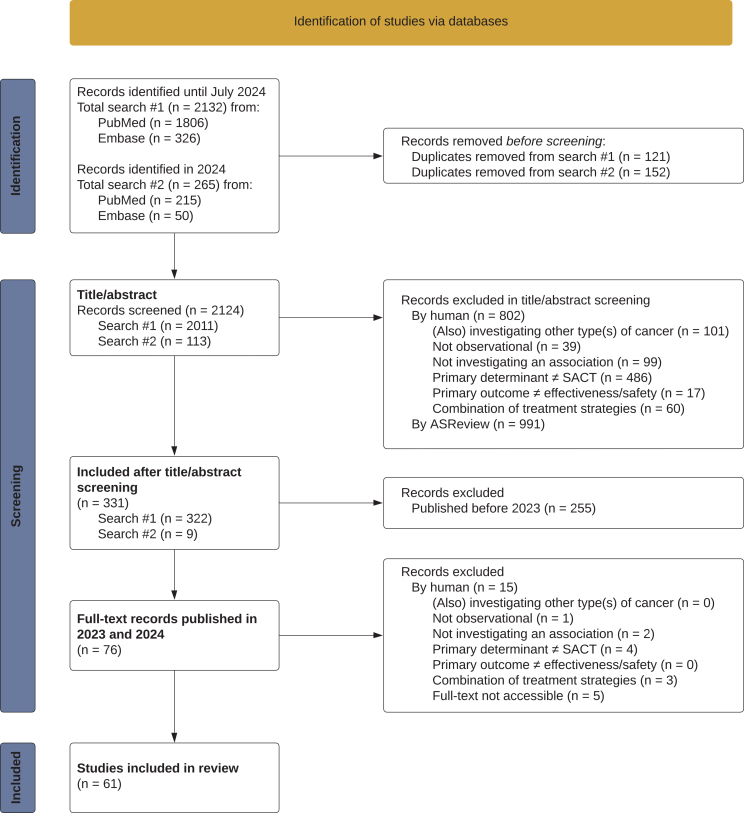
**PRISMA flow diagram.** PRISMA, Preferred Reporting Items for Systematic Reviews and Meta-analyses; SACT, systemic anticancer therapy.

The initial search yielded a total of 2124 records after deduplication (Figure 1). We excluded 802 records through manual review, while 991 records were excluded by ASReview during multiple title/abstract screening phases. We marked 331 records as potentially relevant. We excluded 255 of the 331 relevant records as these were published before 2023. We excluded 15 of the remaining 76 records after full-text screening. Hence, we included 61 RWE studies (34 published in 2023 and 27 published in 2024) in this scoping review.

### Study characteristics

About a third of the RWE studies (31.1%) were published in an oncology journal, while 62.3% were published in another journal category. Of all RWE studies, 59% were published in a Q1 journal, and 65.6% were published open access. The median journal IF was 3.0 (IQR 2.4-3.9, *n* = 59) and the median number of citations was 2 (IQR 1-4, *n* = 57).

Most RWE studies were conducted in Asia (39.3%) and had at least effectiveness as the clinical outcome of interest (90.2%) (Table 2). The most common study setting was the hospital setting (55.7%). The majority of RWE studies used a single data source (47.5%), most often a hospital data source (59%). Approximately a third of the RWE studies (34.4%) reported on the use of guidelines for observational studies. Exposure-based inclusion was often defined by (incident) use (27.9%) or the number of cycles (18%). If reported, the origin of exposure was drug administration data in 36.1% of the RWE studies.

**Table 2:** Characteristics of the included real-world evidence studies (*n* = 61)

Study characteristics	RWE studies	
	%	*n*
Continent		
Asia	39.3	24
Europe	23.0	14
North America	29.5	18
South America	4.9	3
Multicontinent	3.3	2
Clinical outcomes		
Effectiveness	59.0	36
Safety	9.8	6
Both effectiveness and safety	31.2	19
Setting		
Primary care	0.0	0
Hospital	55.7	34
Multiple	6.6	4
Not clear	19.7	12
Not reported	18.0	11
Data source^a^		
Administrative/claims data	21.3	13
General practitioner’s data	0.0	0
Hospital data	59.0	36
Pharmacy data	3.3	2
Cancer registry data	16.4	10
Other	6.6	4
Not clear	4.9	3
Not reported	21.3	13
Reported use of guidelines for observational studies		
Yes	34.4	21
No	65.6	40
Exposure-based inclusion criterion		
No. of administrations	11.5	7
No. of cycles	18.0	11
No. of days exposed	3.3	2
(Cumulative) dose	0.0	0
(Incident) use	27.9	17
Other	6.6	4
Not reported	32.8	20
No. included patients, median (IQR)	211	102-1277
Start of follow-up		
Diagnosis	3.3	2
Exposure	23.0	14
Both diagnosis and exposure	1.6	1
Other	14.8	9
Not reported	57.4	35
Follow-up time (months), median (IQR)	17.2	9.9-33.6
Reported	55.7	34
Not reported	44.3	27
Origin of exposure^b^		
Drug administration data	36.1	22
Drug dispensation data	0.0	0
Drug preparation data	0.0	0
Drug prescription data	11.5	7
Other data	0.0	0
Not clear	42.6	26
Not reported	14.8	9
Treatment type		
Monotherapy	11.5	7
Combination therapy	41.0	25
Both monotherapy and combination therapy	26.2	16
Not clear	14.8	9
Not reported	6.6	4
Type of systemic anticancer therapy		
Chemotherapy	32.8	20
Targeted therapy	16.4	10
Both chemotherapy and targeted therapy	50.8	31

IQR, interquartile range; RWE, real-world evidence.

a Adds up to >100%: 47.5% (29 RWE studies) used a single data source and 26.2% (16 RWE studies) used multiple data sources.

b Adds up to >100%: 37.7% (23 RWE studies) used a single origin of exposure and 4.9% (3 RWE studies) used multiple origins of exposure.

### Reporting of drug exposure definitions

The active substance under study was explicitly reported in the methods section or referenced protocol and/or previous RWE study in 57.4% and was deducible from the regimen in 24.6% of the RWE studies (Table 3). The start of drug exposure was defined in 83.6%. The dose was fully reported in 27.9%, the dosing schedule in 21.3%, and the number of cycles in 8.2%. The treatment episode length and the end of drug exposure were both reported in 34.4% of the RWE studies.

**Table 3:** Reporting of components and variables for oncological drug exposure in real-world evidence studies (*n* = 61)

Key components of oncological drug exposure	RWE studies	
	%	*n*
Active substance(s) reported		
Yes	57.4	35
Partially	24.6	15
No	18.0	11
Start of drug exposure reported		
Yes	83.6	51
No	16.4	10
Dose reported		
Yes	27.9	17
Partially	11.5	7
No	60.7	37
Dosing schedule reported		
Yes	21.3	13
Partially	18.0	11
No	60.7	37
No. cycles reported		
Yes	8.2	5
Partially	19.7	12
No	72.1	44
Treatment episode length reported		
Yes	34.4	21
No	65.6	40
End of drug exposure reported		
Yes	34.4	21
No	65.6	40
Additional variables contributing to oncological drug exposure		
Regimen reported		
Yes	41.0	25
Partially	59.0	36
No	0.0	0
Route of administration reported		
Yes	23.0	14
Partially	16.4	10
No	60.7	37
Treatment switching reported		
Yes	13.1	8
No	86.9	53
Dose adjustment reported		
Yes	21.3	13
No	78.7	48
Cycle prolongation reported		
Yes	8.2	5
No	91.8	56
Bridging between gaps reported		
Yes	4.9	3
No	95.1	58

RWE, real-world evidence.

The regimen was fully reported in 41% and partially reported in 59% of the RWE studies. The route of administration was fully reported in 23% and partially reported in 16.4% of the RWE studies. Definitions for treatment switching, dose adjustments, and cycle prolongation were provided in 13.1%, 21.3%, and 8.2% of the RWE studies, respectively. Three RWE studies (4.9%) accounted for bridging.

### Drug exposure reporting score

**Figure 2:**
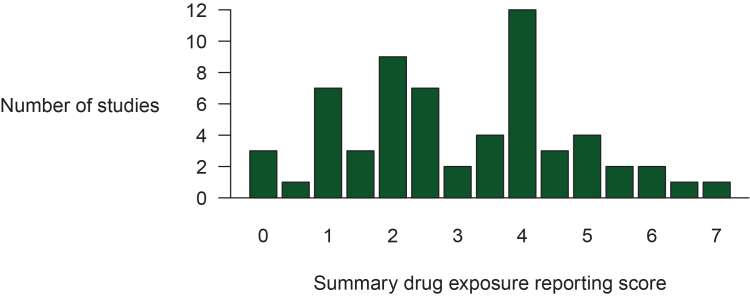
Distribution of the drug exposure reporting score.

The drug exposure reporting score summarizes the reporting of all seven key drug exposure variables, namely the active substance, start of drug exposure, dose, dosing schedule, number of cycles, length of treatment episode, and the end of drug exposure. The median drug exposure reporting score was 3.0 (IQR 2.0-4.0). One RWE study (1.6%), namely the study by Mochizuki et al.^25^, reported all key drug exposure variables, represented by a drug exposure reporting score of 7 (Figure 2). Three RWE studies reported no key drug exposure variables, represented by a drug exposure reporting score of 0.^26^, ^27^, ^28^ Most RWE studies (19.7%, *n* = 12) had a drug exposure reporting score of 4.

Finally, we exploratively compared publication and study characteristics of RWE studies within the highest quartile of the drug exposure reporting score (i.e. score 4.5-7) versus the lowest quartile (i.e. score 0-1.5) (Table 4). Studies with a high drug exposure reporting score were often conducted in Europe (38.5%), and studies with a low drug exposure reporting score were often conducted in Asia and North America (both 42.9%). RWE studies with a high drug exposure reporting score were 4.6 (95% CI 0.6-34.1) times more often published in an oncology journal than RWE studies with a low drug exposure reporting score (38.5% versus 7.1%, respectively). RWE studies with a high drug exposure reporting score were published in a Q1 journal approximately equally often (RR 0.9, 95% CI 0.5-1.5) as RWE studies with a low drug exposure reporting score (61.5% versus 71.4%, respectively). RWE studies with a high drug exposure reporting score were published open access approximately equally often (RR 1.1, 95% CI 0.6-2.0) as RWE studies with a low drug exposure reporting score (61.5% and 57.1%, respectively). The median journal IF (as of 2024) was 2.8 (IQR 2.3-3.4) for high-scoring RWE studies and 3.1 (IQR 2.8-3.8) for low-scoring RWE studies. The median number of citations was 2.0 (IQR 1.0-3.0) for high-scoring RWE studies and 1.0 (IQR 1.0-4.5) for low-scoring RWE studies. RWE studies with a high drug exposure reporting score reportedly used guidelines for observational studies, 1.8 (95% CI 0.5-6.1) times more often than RWE studies with a low drug exposure reporting score (38.5% and 21.4%, respectively).

**Table 4:** A comparison of the publication and study characteristics of RWE studies with a high (4.5-7, *n* = 13) and low (0-1.5, *n* = 14) drug exposure reporting score

	High drug exposure reporting score		Low drug exposure reporting score		Relative risk	95% CI
	%	*n* = 13	%	*n* = 14		
Oncology journal category	38.5	5	7.1	1	4.6	0.6-34.1
NA	0.0	0	14.3	2	—	—
Q1 journal quartiles	61.5	8	71.4	10	0.9	0.5-1.5
Open access	61.5	8	57.1	8	1.1	0.6-2.0
Journal impact factor (2024), median (IQR)	2.8	2.3-3.4	3.1	2.8-3.8	—	—
NA	0.0	0	7.1	1	—	—
No. citations, median (IQR)	2.0	1.0-3.0	1.0	1.0-4.5	—	—
NA	0.0	0	14.3	2	—	—
Reported use of guidelines for observational studies	38.5	5	21.4	3	1.8	0.5-6.1

CI, confidence interval; IQR, interquartile range; RWE, real-world evidence.

## Discussion

This scoping review found that definitions of drug exposure components were often underreported in RWE studies on oncology medicines, published in 2023 and 2024. The start of drug exposure was reported in the majority (83.6%) of RWE studies, whereas the end of drug exposure was reported in only 34.4%. Further, RWE studies often did not report other essential oncological drug exposure components, especially dose, dosing schedule, the number of cycles, and the treatment episode length.

Our findings are in line with those of a previous study, which showed that the quality of reporting drug exposure in observational studies in therapeutic areas beyond oncology was also substandard.^29^ Conceptual details on the reporting of drug exposure were frequently mentioned; all observational studies reported on the type of drug exposure, 85% reported on the drug exposure risk window, and 98% reported on the drug exposure assessment. However, operational details were often underreported; handling gaps between subsequent drug prescriptions or dispensations was reported in 41% of the observational studies, and handling overlaps between drug prescriptions or dispensations in 11%. The underreporting of drug exposure components could partially be explained by unavailable or unstandardized drug exposure components in the used RWD source. In such cases, the data quality and its effect on the exposure–outcome association should be addressed.

It is important to clearly report on drug exposure. Exposure misclassification and large variations in observed outcomes can be the result of different choices in longitudinally modeled drug exposure and constructed drug treatment episodes, using RWD. This was previously shown for simple regimens (e.g. one tablet per day).^30^, ^31^, ^32^ Compared with those simple regimens, constructing oncological treatment episodes is even more complex due to the intricate nature of the treatment itself (i.e. the cyclic oral and/or parenteral administration of one or multiple medicines that make up a regimen, on one or multiple days with rest days in between).

Additional oncology-specific aspects important for clinical interpretation are the line of therapy, regimen-based exposure, and dose intensity. While reporting these additional oncology-specific considerations is important for the interpretation of clinical outcomes, this scoping review primarily focused on the dimensions of individual drug exposure components in the context of constructing oncological treatment episodes from RWD.

Accurately modeling and reporting oncological drug exposure is needed to obtain valid estimates of the association between oncology medicines and clinical outcomes in RWE studies. Underreporting of oncological drug exposure does not necessarily translate into poor methodological quality, but it does challenge the assessment of the methodological quality of a study as well as the interpretation of findings and reproducibility. Regulators and clinicians increasingly use RWE to supplement their decision making regarding marketing authorization, reimbursement, and/or uptake in clinical guidelines of new and often expensive oncology medicines.^33^, ^34^, ^35^ The potential consequences of exposure misclassification and biased estimates emphasize the importance of adequately defining and reporting oncological drug exposure in RWE studies.

In this scoping review, we selected the components that are minimally required for accurately modeling exposure to oncology medicines: active substance, start of drug exposure, dose, dosing schedule, number of cycles, treatment episode length, and end of drug exposure. Existing RWE reporting guidelines do not completely capture the complexity of oncological drug exposure.^12^ Hence, we created the drug exposure reporting score to assess the extent to which key components of oncological drug exposure were reported in RWE studies. The halved weight of components that were partially reported reflects the uncertainty of having to deduce information. It thus reflects how many of the key components of oncological drug exposure were (partially) reported, with each component given equal weight. The median drug exposure reporting score was 3.0 (IQR 2.0-4.0), with only one RWE study fully reporting all seven key drug exposure components.^25^ The quality of reporting non-oncological drug exposure in observational studies was assessed through the reporting of 11 conceptual and operational items defined by the International Society of Pharmacoepidemiology (ISPE)-International Society for Pharmacoeconomics and Outcomes Research (ISPOR) Joint Task Force guideline.^29^^,^^36^ These items show little overlap with the key drug exposure components selected in our study. Both studies assessed treatment switching and bridging between gaps. We found that RWE studies on oncology medicines reported treatment switching in 13.1% and bridging between gaps in only 4.9%. For reference, observational studies on non-oncological drug exposure reported treatment switching in 62% and bridging between gaps in 41%.

After explorative analysis, we found that RWE studies with a high drug exposure reporting score were 4.6 times more likely to be published in an oncology journal than RWE studies with a low drug exposure reporting score, although not statistically significant. A potential explanation for this could be that oncology journals value the clear reporting of oncological drug exposure more than other journal types. RWE studies with a high drug exposure reporting score were 1.8 times more likely to report on the use of guidelines for observational studies than RWE studies with a low drug exposure reporting score, although not statistically significant. Guidelines for observational studies recommend reporting practices in observational studies, thereby improving the quality of reporting.^37^ This encourages authors to explicitly report exposure. It should be kept in mind that some study characteristics might inherently be related to each other. These exploratory results should therefore be interpreted in light of such dependencies, the relatively small sample sizes, and the broad CIs.

This scoping review has several strengths. To the best of our knowledge, this is the first review on the reporting of oncological drug exposure in RWE studies. We systematically searched for eligible RWE studies and identified relevant RWE studies through an efficient and robust, multiphase screening process, aided by ASReview. Some relevant records might have been missed regardless of the systematic search, but the potentially missed articles are not expected to change the direction of the results. Another strength is the descriptive and exploratory comparison between RWE studies with a high and low drug exposure reporting score, which suggests what factors might be related to the extent to which oncological drug exposure is reported (i.e. publication in an oncology journal and reported use of guidelines for observational studies).

A limitation is that the scope is restricted by time and tumor type. We focused on RWE studies most relevant for today’s decision making and clinical practice by narrowing down the scope to 2023 and 2024. We also focused on RWE studies in CRC because its treatment contains multiple complex exposure aspects, such as cyclic administration, both orally and intravenously. Regardless of the restrictions, the findings of this scoping review are expected to be generalizable to other malignancies with similar treatment complexity. Although restricted, the scope is deemed sufficient to identify gaps in the reporting of exposure to oncological medicines in general.

Another limitation is the fact that we looked at the reporting of drug exposure in the methods section of RWE studies only. Some key drug exposure components might not have been mentioned in the methods section but can be deduced with clinical knowledge. For instance, the active substances, and sometimes also the dosing schedule, number of cycles, and treatment episode length, can be deduced from the regimen name. Such deductions are uncertain due to geographical and temporal differences and dependency on other drug exposure components (e.g. treatment episode length may be influenced by cycle prolongation). Additionally, some RWE studies did mention summary statistics of drug exposure variables (e.g. dose) in the results section. This indicates that the authors assessed those exposure variables, but information on their definition is lacking. As a result, the transparency and reproducibility of RWE studies are impeded.^36^ This can be improved by thoroughly reporting oncological drug exposure in the methods section of RWE studies, thereby reinforcing the validity of the RWE results. In the current study, we gave equal weight to each element of the drug exposure reporting score. However, not all element may be equally important for the reproducibility of an RWE study and the interpretation of findings. Future studies should validate the drug exposure reporting score and evaluate whether individual oncological drug exposure components vary in importance for different clinical research questions.

## Conclusion

Publications on RWE studies underreport definitions of oncological drug exposure, hampering the interpretation of the potential associations between oncology medicines and clinical outcomes. We recommend authors provide a detailed report in the methods section on how (at least the key) drug exposure components of oncology medicines were defined in RWE studies. Authors are advised to use the drug exposure reporting score to assess the comprehensiveness of their reporting. This is expected to improve the validity of the estimated association and substantiate decision making regarding marketing authorization, reimbursement, and uptake of oncology medicines in clinical guidelines. As a result, new and often expensive oncology medicines can be used more rationally.
