# Supplementary material for: Drug exposure definitions reported in real-world evidence studies on oncology medicines in patients with colorectal cancer: a scoping review
Source: ESMO Real World Data Digit Oncol. 2026 Aug 10;13:100750. doi: 10.1016/j.esmorw.2026.100750 (PMC13486887; doi:10.1016/j.esmorw.2026.100750)
Supplement: Supplementary file 1 [file mmc1.docx]

## Supplementary material for:

## Drug exposure definitions reported in real-world evidence studies on oncology medicines in colorectal cancer patients: a scoping review

ESMO Real World Data and Digital Oncology

**Table S1** Search string in PubMed and Embase

| **1) (metastatic) colorectal cancer** | **2) Systemic anticancer treatment** | **3) Observational study design** | **4) Observational data source** | **5) Publication type** |
| --- | --- | --- | --- | --- |
| (colorectal neoplasm[MeSH Terms]) OR (colorectal neoplasm*[Title/Abstract]) OR (colorectal carcinom* [Title/Abstract]) OR (colorectal cancer[Title/Abstract]) OR (colorectal tumo*[Title/Abstract]) OR (metastatic colorectal neoplasm*[Title/Abstract]) OR (metastatic colorectal carcinom* [Title/Abstract]) OR (metastatic colorectal cancer[Title/Abstract]) OR (metastatic colorectal tumo*[Title/Abstract]) | (agents, antineoplastic[MeSH Terms]) OR (adjuvant chemotherapy[MeSH Terms]) OR (neoadjuvant therapy[MeSH Terms]) OR (antineoplastic protocol[MeSH Terms]) OR (molecular targeted therapy[MeSH Terms]) OR (chemotherap*[Title/Abstract]) OR (systemic anticancer therap*[Title/Abstract]) OR (systemic anti-cancer therap*[Title/Abstract]) OR (SACT[Title/Abstract]) OR (targeted therap*[Title/Abstract]) OR (immunotherap*[Title/Abstract]) OR (antineoplastic agent*[Title/Abstract]) OR (antineoplastic therap*[Title/Abstract]) OR (antineoplastic drug*[Title/Abstract]) OR (antineoplastic treatment*[Title/Abstract]) OR (oncolytic agent*[Title/Abstract]) OR (oncolytic therap*[Title/Abstract]) OR (oncolytic drug*[Title/Abstract]) OR (oncolytic treatment*[Title/Abstract]) OR (antineoplastic regimen*[Title/Abstract]) OR (oncolytic regimen*[Title/Abstract]) OR (anticancer agent*[Title/Abstract]) OR (anticancer therap*[Title/Abstract]) OR (anticancer drug*[Title/Abstract]) OR (anticancer treatment*[Title/Abstract]) OR (anti-cancer agent*[Title/Abstract]) OR (anti-cancer therap*[Title/Abstract]) OR (anti-cancer drug*[Title/Abstract]) OR (anti-cancer treatment*[Title/Abstract]) OR (anti-cancer regimen*[Title/Abstract]) OR (anticancer regimen*[Title/Abstract]) OR (oncolytic regimen*[Title/Abstract]) | (case control study[Title/Abstract]) OR (case-control study[Title/Abstract]) OR (case crossover study[Title/Abstract]) OR (case-crossover study[Title/Abstract]) OR (cohort study[Title/Abstract]) | (pharmacy claims[Title/Abstract]) OR (pharmacy data[Title/Abstract]) OR (dispensing data[Title/Abstract]) OR (computerized data[Title/Abstract]) OR (computerized database[Title/Abstract]) OR (administrative claims[Title/Abstract]) OR (prescription claims[Title/Abstract]) OR (prescription database[Title/Abstract]) OR (prescription data[Title/Abstract]) OR (health care database[Title/Abstract]) OR (health database[Title/Abstract]) OR (health care claims[Title/Abstract]) OR (electronic health record*[Title/Abstract]) OR (insurance plan[Title/Abstract]) OR (medicaid[Title/Abstract]) OR (managed care organization[Title/Abstract]) OR (veterans affairs[Title/Abstract]) | (Observational study[Publication Type]) |
| Seach string PubMed:  ( (colorectal neoplasm[MeSH Terms]) OR (colorectal neoplasm*[Title/Abstract]) OR (colorectal carcinom* [Title/Abstract]) OR (colorectal cancer[Title/Abstract]) OR (colorectal tumo*[Title/Abstract]) OR (metastatic colorectal neoplasm*[Title/Abstract]) OR (metastatic colorectal carcinom* [Title/Abstract]) OR (metastatic colorectal cancer[Title/Abstract]) OR (metastatic colorectal tumo*[Title/Abstract]) ) AND ( (agents, antineoplastic[MeSH Terms]) OR (adjuvant chemotherapy[MeSH Terms]) OR (neoadjuvant therapy[MeSH Terms]) OR (antineoplastic protocol[MeSH Terms]) OR (molecular targeted therapy[MeSH Terms]) OR (chemotherap*[Title/Abstract]) OR (systemic anticancer therap*[Title/Abstract]) OR (systemic anti-cancer therap*[Title/Abstract]) OR (SACT[Title/Abstract]) OR (targeted therap*[Title/Abstract]) OR (immunotherap*[Title/Abstract]) OR (antineoplastic agent*[Title/Abstract]) OR (antineoplastic therap*[Title/Abstract]) OR (antineoplastic drug*[Title/Abstract]) OR (antineoplastic treatment*[Title/Abstract]) OR (oncolytic agent*[Title/Abstract]) OR (oncolytic therap*[Title/Abstract]) OR (oncolytic drug*[Title/Abstract]) OR (oncolytic treatment*[Title/Abstract]) OR (antineoplastic regimen*[Title/Abstract]) OR (oncolytic regimen*[Title/Abstract]) OR (anticancer agent*[Title/Abstract]) OR (anticancer therap*[Title/Abstract]) OR (anticancer drug*[Title/Abstract]) OR (anticancer treatment*[Title/Abstract]) OR (anti-cancer agent*[Title/Abstract]) OR (anti-cancer therap*[Title/Abstract]) OR (anti-cancer drug*[Title/Abstract]) OR (anti-cancer treatment*[Title/Abstract]) OR (anti-cancer regimen*[Title/Abstract]) OR (anticancer regimen*[Title/Abstract]) OR (oncolytic regimen*[Title/Abstract]) ) AND ( ((case control study[Title/Abstract]) OR (case-control study[Title/Abstract]) OR (case crossover study[Title/Abstract]) OR (case-crossover study[Title/Abstract]) OR (cohort study[Title/Abstract])) OR ((pharmacy claims[Title/Abstract]) OR (pharmacy data[Title/Abstract]) OR (dispensing data[Title/Abstract]) OR (computerized data[Title/Abstract]) OR (computerized database[Title/Abstract]) OR (administrative claims[Title/Abstract]) OR (prescription claims[Title/Abstract]) OR (prescription database[Title/Abstract]) OR (prescription data[Title/Abstract]) OR (health care database[Title/Abstract]) OR (health database[Title/Abstract]) OR (health care claims[Title/Abstract]) OR (electronic health record*[Title/Abstract]) OR (insurance plan[Title/Abstract]) OR (medicaid[Title/Abstract]) OR (managed care organization[Title/Abstract]) OR (veterans affairs[Title/Abstract])) OR ((Observational study[Publication Type])) ) | | | | 1,806 results  29-07-2024 14:20 |
| Search string PubMed (restricted to 2024):  ( (colorectal neoplasm[MeSH Terms]) OR (colorectal neoplasm*[Title/Abstract]) OR (colorectal carcinom* [Title/Abstract]) OR (colorectal cancer[Title/Abstract]) OR (colorectal tumo*[Title/Abstract]) OR (metastatic colorectal neoplasm*[Title/Abstract]) OR (metastatic colorectal carcinom* [Title/Abstract]) OR (metastatic colorectal cancer[Title/Abstract]) OR (metastatic colorectal tumo*[Title/Abstract]) ) AND ( (agents, antineoplastic[MeSH Terms]) OR (adjuvant chemotherapy[MeSH Terms]) OR (neoadjuvant therapy[MeSH Terms]) OR (antineoplastic protocol[MeSH Terms]) OR (molecular targeted therapy[MeSH Terms]) OR (chemotherap*[Title/Abstract]) OR (systemic anticancer therap*[Title/Abstract]) OR (systemic anti-cancer therap*[Title/Abstract]) OR (SACT[Title/Abstract]) OR (targeted therap*[Title/Abstract]) OR (immunotherap*[Title/Abstract]) OR (antineoplastic agent*[Title/Abstract]) OR (antineoplastic therap*[Title/Abstract]) OR (antineoplastic drug*[Title/Abstract]) OR (antineoplastic treatment*[Title/Abstract]) OR (oncolytic agent*[Title/Abstract]) OR (oncolytic therap*[Title/Abstract]) OR (oncolytic drug*[Title/Abstract]) OR (oncolytic treatment*[Title/Abstract]) OR (antineoplastic regimen*[Title/Abstract]) OR (oncolytic regimen*[Title/Abstract]) OR (anticancer agent*[Title/Abstract]) OR (anticancer therap*[Title/Abstract]) OR (anticancer drug*[Title/Abstract]) OR (anticancer treatment*[Title/Abstract]) OR (anti-cancer agent*[Title/Abstract]) OR (anti-cancer therap*[Title/Abstract]) OR (anti-cancer drug*[Title/Abstract]) OR (anti-cancer treatment*[Title/Abstract]) OR (anti-cancer regimen*[Title/Abstract]) OR (anticancer regimen*[Title/Abstract]) OR (oncolytic regimen*[Title/Abstract]) ) AND ( ((case control study[Title/Abstract]) OR (case-control study[Title/Abstract]) OR (case crossover study[Title/Abstract]) OR (case-crossover study[Title/Abstract]) OR (cohort study[Title/Abstract])) OR ((pharmacy claims[Title/Abstract]) OR (pharmacy data[Title/Abstract]) OR (dispensing data[Title/Abstract]) OR (computerized data[Title/Abstract]) OR (computerized database[Title/Abstract]) OR (administrative claims[Title/Abstract]) OR (prescription claims[Title/Abstract]) OR (prescription database[Title/Abstract]) OR (prescription data[Title/Abstract]) OR (health care database[Title/Abstract]) OR (health database[Title/Abstract]) OR (health care claims[Title/Abstract]) OR (electronic health record*[Title/Abstract]) OR (insurance plan[Title/Abstract]) OR (medicaid[Title/Abstract]) OR (managed care organization[Title/Abstract]) OR (veterans affairs[Title/Abstract])) OR ((Observational study[Publication Type])) ) | | | | 240 results  19-06-2024  11:28 |
| Search string EMBASE:  ('colorectal tumor'/exp OR 'colorectal neoplasm*':ti,ab,kw OR 'colorectal carcinom*':ti,ab,kw OR 'colorectal cancer':ti,ab,kw OR 'colorectal tumo*':ti,ab,kw OR 'metastatic colorectal neoplasm*':ti,ab,kw OR 'metastatic colorectal carcinom*':ti,ab,kw OR 'metastatic colorectal cancer':ti,ab,kw OR 'metastatic colorectal tumo*':ti,ab,kw) AND ('agents, antineoplastic' OR 'adjuvant chemotherapy'/exp OR 'neoadjuvant therapy'/exp OR 'antineoplastic protocol'/exp OR 'molecularly targeted therapy'/exp OR 'chemotherap*':ti,ab,kw OR 'systemic anticancer therap*':ti,ab,kw OR 'systemic anti-cancer therap*':ti,ab,kw OR 'sact':ti,ab,kw OR 'targeted therap*':ti,ab,kw OR 'immunotherap*':ti,ab,kw OR 'antineoplastic agent*':ti,ab,kw OR 'antineoplastic therap*':ti,ab,kw OR 'antineoplastic drug*':ti,ab,kw OR 'antineoplastic treatment*':ti,ab,kw OR 'oncolytic agent*':ti,ab,kw OR 'oncolytic therap*':ti,ab,kw OR 'oncolytic drug*':ti,ab,kw OR 'oncolytic treatment*':ti,ab,kw OR 'antineoplastic regimen*':ti,ab,kw OR 'anticancer agent*':ti,ab,kw OR 'anticancer therap*':ti,ab,kw OR 'anticancer drug*':ti,ab,kw OR 'anticancer treatment*':ti,ab,kw OR 'anti-cancer agent*':ti,ab,kw OR 'anti-cancer therap*':ti,ab,kw OR 'anti-cancer drug*':ti,ab,kw OR 'anti-cancer treatment*':ti,ab,kw OR 'anti-cancer regimen*':ti,ab,kw OR 'anticancer regimen*':ti,ab,kw OR 'oncolytic regimen*':ti,ab,kw) AND ('case control study':ti,ab,kw OR 'case-control study':ti,ab,kw OR 'case crossover study':ti,ab,kw OR 'case-crossover study':ti,ab,kw OR 'cohort study':ti,ab,kw OR 'pharmacy claims':ti,ab,kw OR 'pharmacy data':ti,ab,kw OR 'dispensing data':ti,ab,kw OR 'computerized data':ti,ab,kw OR 'computerized database':ti,ab,kw OR 'administrative claims':ti,ab,kw OR 'prescription claims':ti,ab,kw OR 'prescription database':ti,ab,kw OR 'prescription data':ti,ab,kw OR 'health care database':ti,ab,kw OR 'health database':ti,ab,kw OR 'health care claims':ti,ab,kw OR 'electronic health record*':ti,ab,kw OR 'insurance plan':ti,ab,kw OR 'medicaid':ti,ab,kw OR 'managed care organization':ti,ab,kw OR 'veterans affairs':ti,ab,kw OR 'observational study':it)  #1 NOT 'conference abstract'/it  #2 AND [embase]/lim NOT ([embase]/lim AND [medline]/lim) | | | | 326 results  29-07-2024 14:46 |
| Search string EMBASE (restricted to 2024):  ('colorectal tumor'/exp OR 'colorectal neoplasm*':ti,ab,kw OR 'colorectal carcinom*':ti,ab,kw OR 'colorectal cancer':ti,ab,kw OR 'colorectal tumo*':ti,ab,kw OR 'metastatic colorectal neoplasm*':ti,ab,kw OR 'metastatic colorectal carcinom*':ti,ab,kw OR 'metastatic colorectal cancer':ti,ab,kw OR 'metastatic colorectal tumo*':ti,ab,kw) AND ('agents, antineoplastic' OR 'adjuvant chemotherapy'/exp OR 'neoadjuvant therapy'/exp OR 'antineoplastic protocol'/exp OR 'molecularly targeted therapy'/exp OR 'chemotherap*':ti,ab,kw OR 'systemic anticancer therap*':ti,ab,kw OR 'systemic anti-cancer therap*':ti,ab,kw OR 'sact':ti,ab,kw OR 'targeted therap*':ti,ab,kw OR 'immunotherap*':ti,ab,kw OR 'antineoplastic agent*':ti,ab,kw OR 'antineoplastic therap*':ti,ab,kw OR 'antineoplastic drug*':ti,ab,kw OR 'antineoplastic treatment*':ti,ab,kw OR 'oncolytic agent*':ti,ab,kw OR 'oncolytic therap*':ti,ab,kw OR 'oncolytic drug*':ti,ab,kw OR 'oncolytic treatment*':ti,ab,kw OR 'antineoplastic regimen*':ti,ab,kw OR 'anticancer agent*':ti,ab,kw OR 'anticancer therap*':ti,ab,kw OR 'anticancer drug*':ti,ab,kw OR 'anticancer treatment*':ti,ab,kw OR 'anti-cancer agent*':ti,ab,kw OR 'anti-cancer therap*':ti,ab,kw OR 'anti-cancer drug*':ti,ab,kw OR 'anti-cancer treatment*':ti,ab,kw OR 'anti-cancer regimen*':ti,ab,kw OR 'anticancer regimen*':ti,ab,kw OR 'oncolytic regimen*':ti,ab,kw) AND ('case control study':ti,ab,kw OR 'case-control study':ti,ab,kw OR 'case crossover study':ti,ab,kw OR 'case-crossover study':ti,ab,kw OR 'cohort study':ti,ab,kw OR 'pharmacy claims':ti,ab,kw OR 'pharmacy data':ti,ab,kw OR 'dispensing data':ti,ab,kw OR 'computerized data':ti,ab,kw OR 'computerized database':ti,ab,kw OR 'administrative claims':ti,ab,kw OR 'prescription claims':ti,ab,kw OR 'prescription database':ti,ab,kw OR 'prescription data':ti,ab,kw OR 'health care database':ti,ab,kw OR 'health database':ti,ab,kw OR 'health care claims':ti,ab,kw OR 'electronic health record*':ti,ab,kw OR 'insurance plan':ti,ab,kw OR 'medicaid':ti,ab,kw OR 'managed care organization':ti,ab,kw OR 'veterans affairs':ti,ab,kw OR 'observational study':it)  #1 NOT 'conference abstract'/it  #2 AND [embase]/lim NOT ([embase]/lim AND [medline]/lim) AND [2024-2024]/py | | | | 50 results  19-06-2025  11:37 |

**Table S2** Data extraction template

| **Variable** | **Specification** |
| --- | --- |
| Study ID | Free text |
| First author | Free text |
| Title | Free text |
| Publication year | Free text |
| Publication journal | Free text |
| Journal category | Free text + Oncology / Other / NA |
| Journal quartile | Q1 / Q2 / Q3 / Q4 / NA |
| Open access | Yes / No |
| Journal impact factor | Free text |
| Number of citations | Free text |
| Reported use of guidelines for observational studies | Free text + Yes / No |
| Country | Free text |
| Continent | Africa / Asia / Europe / North America / Oceania South America / Multi-continent / Not reported |
| Clinical primary outcome | Free text + Effectiveness / Safety / Both |
| Follow-up time (in months) | Free text |
| Number of included subjects | Free text |
| Study setting | Free text + Primary / Hospital / Multiple / Other / Not clear / Not reported |
| Data source | Free text + Administrative/claims database / General practitioners’ database / Hospital database / Pharmacy database / Registry / Multiple / Other / Not clear / Not reported |
| Origin of exposure data | Free text + Drug administration data / Drug dispensation data / Drug preparation data / Drug prescription data / Other / Multiple / Not clear /Not reported |
| Exposure-based inclusion criterium | Free text + Number of administrations / Number of cycles / (Cumulative) dose / Number of days exposed / (Incident) use / Other / Not clear / Not reported |
| Treatment type | Monotherapy / Combination therapy / Both mono- and combination therapy / Not clear / Not reported |
| Regimen | Free text + Reported / Partially reported / Not reported |
| Active substance | Free text + Reported / Partially reported / Not reported |
| Dose | Free text + Reported / Partially reported / Not reported |
| Number of cycles | Free text + Reported / Partially reported / Not reported |
| Dosing schedule | Free text + Reported / Partially reported / Not reported |
| Route of administration | Intravenous / Oral / Both / Not clear / Not reported |
| Start of follow-up | Diagnosis / Exposure / Other / Not clear / Not reported |
| Start of drug exposure | Free text + Reported / Not reported |
| End of drug exposure | Free text + Reported / Not reported |
| Treatment episode length definition | Free text + Reported / Not reported |
| Treatment switch definition | Free text + Reported / Not reported |
| Dose alteration definition | Free text + Reported / Not reported |
| Cycle prolongation definition | Free text + Reported / Not reported |
| Bridging definition | Free text + Reported / Not reported |
| Graphical representation of exposure | Reported / Not reported |
